# Assessing the learning curve associated with a novel flexible robot in the pre-clinical and clinical setting

**DOI:** 10.1007/s00464-021-08445-7

**Published:** 2021-03-22

**Authors:** Toby S. Zhu, Neal Godse, Daniel R. Clayburgh, Umamaheswar Duvvuri

**Affiliations:** 1 University of Pittsburgh School of Medicine, Pittsburgh, PA USA; 2 Department of Otolaryngology, University of Pittsburgh Medical Center, Eye and Ear Institute, Suite 500, 200 Lothrop St, Pittsburgh, PA USA; 3 Department of Otolaryngology–Head and Neck Surgery, Oregon Health and Science University, Portland, USA; 4 Portland Veterans Affairs Medical Center, Portland, USA

**Keywords:** Medrobotics, FLEX, Transoral robotic surgery, TORS, Learning curve, Training

## Abstract

**Background:**

Transoral robotic surgery has been successfully used by head and neck surgeons for a variety of procedures but is limited by rigid instrumentation and line-of-sight visualization. Non-linear systems specifically designed for the aerodigestive tract are needed. Ease of use of these new systems in both training and clinical environments is critical in its widespread adoption.

**Methods:**

Residents, fellows, and junior faculty performed four tasks on an anatomical airway mannequin using the Medrobotics FLEX™ Robotic System: expose and incise the tonsil, grasp the epiglottis, palpate the vocal processes, and grasp the interarytenoid space. These tasks were performed once a day for four days; after a 4-month time gap, subjects were asked to perform these same tasks for three more days. Time to task completion and total distance driven were tracked. In addition, a retrospective analysis was performed analyzing one attending physician’s experience with clinical usage of the robot.

**Results:**

13 subjects completed the initial round of the mannequin simulation and 8 subjects completed the additional testing 4 months later. Subjects rapidly improved their speed and efficiency at task completion. Junior residents were slower in most tasks initially compared to senior trainees but quickly reached similar levels of efficiency. Following the break there was minimal degradation in skills and continued improvement in efficiency was observed with additional trials. There was significant heterogeneity in the analyzed clinical cases, but when analyzing cases of similar complexity and pathology, clear decreases in overall operative times were demonstrable.

**Conclusion:**

Novice users quickly gained proficiency with the FLEX™ Robotic System in a training environment, and these skills are retained after several months. This learning could translate to the clinical setting if a proper training regimen is developed. The Medrobotics FLEX™ Robotic System shows promise as a surgical tool in head and neck surgery in this study.

**Supplementary Information:**

The online version contains supplementary material available at 10.1007/s00464-021-08445-7.

Treatment of head and neck cancer has evolved over the past decade to allow for minimally invasive surgeries that reduce the significant patient morbidity and mortality that accompany traditional approaches [1]. The adoption of transoral robotic surgery (TORS) has allowed surgeons to control endoscopic instruments through the oral cavity and has become widely adopted for numerous otolaryngologic procedures. Prior studies on TORS in head and neck cancer have shown its feasibility [2] and similar oncologic outcomes to traditional methods [3]. The most commonly used surgical platform, the da Vinci robotic system (Intuitive Surgical, Sunnyvale CA), allows for three-dimensional visualization, a magnified field of view for smaller operations, and reduction of physiologic tremors. Although this system is currently being used to great success transorally, it was originally adopted for operations in the thorax and abdomen. The system relies on long, rigid instrument arms that can navigate the truncal anatomy but has issues in the non-linear confines of the aerodigestive tract. Patients with complicated anatomy that prevents linear, line-of-sight exposure of pathology are unable to benefit from the minimally invasive nature of TORS.

This unmet need is addressed by the Medrobotics FLEX™ System, which has a single, flexible robotic arm with attached cameras and instruments. This flexible robot was designed to work in the confines of the upper aerodigestive tract, as it can navigate in a twisted, non-linear path while simultaneously providing a stable base to perform procedures. More detailed design characteristics [4], efficacy in cadavers [5], and usage in humans [6] have been previously described. Adoption of any innovative technique or device by a diverse user base requires an assessment of the learning curve. It is unknown if and how quickly novice users would be able to improve performance while using a non-linear system, like the FLEX system. This study aimed to identify the characteristics and time frame of performance optimization using the FLEX system, analyze the retention or degradation of skills after periods of non-use, and retrospectively review clinical performance of the FLEX robot system in cases from 2015 to 2020.

## Materials and methods

### Subjects

For the mannequin simulation section of this study, 13 subjects were recruited from the otolaryngology residents, fellows, and junior faculty at the University of Pittsburgh for the initial training period. The subjects were divided into 3 groups based on seniority: junior residents (PGY2 and 3), senior residents (PGY4 and 5), and fellows/junior faculty. 8 subjects went on to complete the additional tasks 4 months later. This study was approved by the institutional review board of the University of Pittsburgh.

For the clinical aspect of this study, a retrospective analysis was performed by a chart review of a single attending’s first 20 patients who had surgery using the FLEX™ Robot. Institutional review board approval was also obtained. Cases where robotic usage was attempted but then aborted were excluded.

### Experimental setup

The Medrobotics FLEX™ Robot System (Medrobotics, Raynham, MA) consists of a computer-driven flexible endoscope and has been previously described [1, 4, 5]. Briefly, this system comprises 50 discrete, cylindrical linkages that can rotate about each other. The lead linkage has a video camera, LED lamps, and three instrument channels, all of which are controlled by the surgeon. The endoscope mount also has two working ports where many different flexible instruments can be inserted and used in the operative field.

**Fig. 1:**
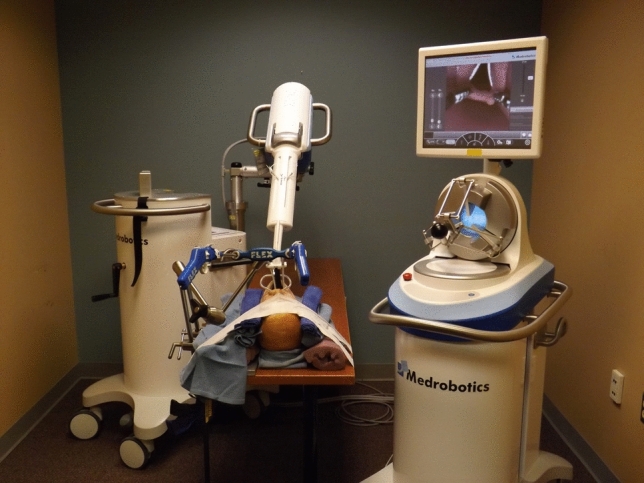
The Medrobotics FLEX™ System and experimental setup. The flexible endoscope device can be seen in white and is operated by the surgeon from a computer station fitted with a haptic drive control. There are also two working ports where various surgical instruments can be brought into the operating field and are directly manipulated by the surgeon standing at the head of the bed. This setup allows for flexible access into the upper aerodigestive tract with one primary scope. The airway mannequin head can also be seen secured to the table

In the mannequin simulation section of this study, a grasping instrument similar to a Maryland dissector and a spatula-shaped electrocautery device were used. Trials were conducted using an anatomic airway mannequin (Life/form “Airway Larry” Adult airway management trainer head) that consisted of a model head and neck with standard pharyngeal and laryngeal anatomy. After the model was secured to the table, a Dingman retractor was inserted into the mannequin’s oral cavity to retract open the mouth and expose the oropharynx and larynx for surgical access. The FLEX™ System was then secured and positioned for transoral access above the head. The end of the endoscopic arm was positioned at the level of the incisors in the midline; for measurement and data analysis purposes, this position was designated as the origin where (x_0_, y_0_, z_0_) = (0, 0, 0). After proper positioning, the robotic system was driven into the pharynx several times to verify that all areas of the pharynx and larynx were accessible, and that the system was functional. The experimental setup with the docked robot is shown in Fig. 1.

### Surgical tasks

In the mannequin simulation section of this study, subjects were asked to perform a series of four surgical tasks on the mannequin. These tasks included the following: (1) tonsil task––exposing the right tonsil, grasping the anterior tonsillar pillar, and tracing a tonsillectomy incision; (2) epiglottis task––exposing the epiglottis, grasping the epiglottis, and touching an electrocautery instrument to the tip of the epiglottis; (3) vocal process task––exposing the glottis and touching the vocal processes bilaterally; and (4) interarytenoid task: exposing the glottis, grasping the interarytenoid area, and touching an electrocautery instrument to the area. Each task consisted of two phases: a drive phase, where the endoscopic arms are driven from the origin position to the appropriate position depending on the task, and an instrument phase, where the two surgical instruments are deployed through the working ports and performance of the designated surgical task. Subjects performed all four tasks during teach testing session, and each subject underwent four testing sessions on four different days over the course of a week. After a four-month period without using the device, subjects were again asked to perform the same tasks with the FLEX™ Robot to measure learning retention over time. The 5 subjects who did not proceed with the second portion of the study were excluded in the retention analysis.

In the clinical aspect of this study, a heterogeneous mix of operations were performed (Supplemental Table S1). The Flex system was used to visualize and manipulate tissue at all subsides of the upper aero digestive tract and case complexity ranged from excision of simple supraglottic lesions to complex, multistage oncologic surgeries.

### Data collection

For the mannequin simulation section of this study, subjects were timed during the performance of each task, starting from the beginning of the drive phase to the completion of the instrument phase. The overall movement of the head of the FLEX™ system was tracked using an Ascension Magnetic trakSTAR with Model 180 (2.0 mm) sensor (Ascension Technology Corporation, Shelburne, VT) attached to the endoscopic arm. The position of the arm was recorded in x, y, and z coordinates at a 60 Hz sampling rate (every 0.0167 s). Movement tracking was only performed during the drive phase of each task.

In the clinical aspect of this study, the surgical start and stop times from the anesthesia record were used to determine total surgical time. The clinical setting precluded use of the non-clinical magnetic tracking system, so trajectory data could not be collected. When applicable, details from the pathology report, such as final diagnosis, grading, and staging, were extracted. For data analysis purposes, a subset of the first 20 cases that were considered “simple” (e.g. excluding neck dissections, cases where frozen samples were sent for margin analysis during the procedure, and cases with a multitude of subcomponents) with benign pathology were extracted.

### Data analysis

In the mannequin simulation section of this study, total time for the drive phase was derived from the tracker data, and time for the instrument phase was obtained by subtracting the total time by the drive phase time. The total distance traveled by the tip of the endoscope was calculated from the positional tracker data using the following formula:

$$D_{total} = \mathop \sum \limits_{t = 0}^{n} \left| {x_{t + 1} - x} \right| + \mathop \sum \limits_{t = 0}^{n} \left| {y_{t + 1} - y} \right| + \mathop \sum \limits_{t = 0}^{n} \left| {z_{t + 1} - z} \right|$$

where D_total_ is the total distance traveled, x_t_, y_t,_ and z_t_ represent the x, y, and z positions of the tracker probe at time t, and n is the number of timepoints in that drive phase. Descriptive statistics were performed on each variable. Changes in total time and total distance traveled were compared between groups using ANOVA testing. The overall speed was calculated by dividing the total distance traveled by the task completion time. To assess the accuracy of the recorded position data, the path length for each trial was measured by marking the starting and ending position of the robot. A linear regression analysis was performed using the measured path length and calculated path length from the tracker.

For the clinical aspect of this study, linear regression and R-square calculations were performed with the first 20 surgical cases.

## Results

### Path length validation

**Fig. 2:**
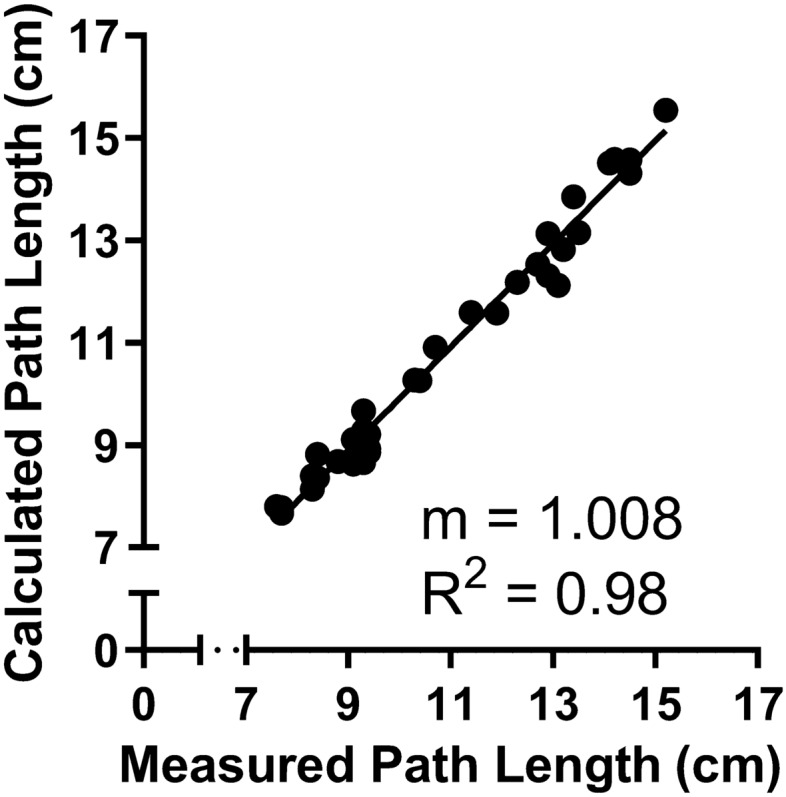
Comparing Measured Path Length and Calculated Path Length. Trials were to vocal folds, epiglottis, and L/R base of tongue. The Measured Path Length was measured after every trial; the Calculated Path Length was calculated from position data. *n* = 37. Trend line is linear regression with slope (m) and R2 as marked

The measured and calculated path lengths were recorded over multiple trials (*n* = 37) as the robot was driven to multiple targets. These two measurements were plotted against each other for each trial and all targets (Fig. 2). The linear regression produced a best-fit line with an R^2^ = 0.9806 and a slope of m = 1.008.

### Mannequin learning trials

**Fig. 3:**
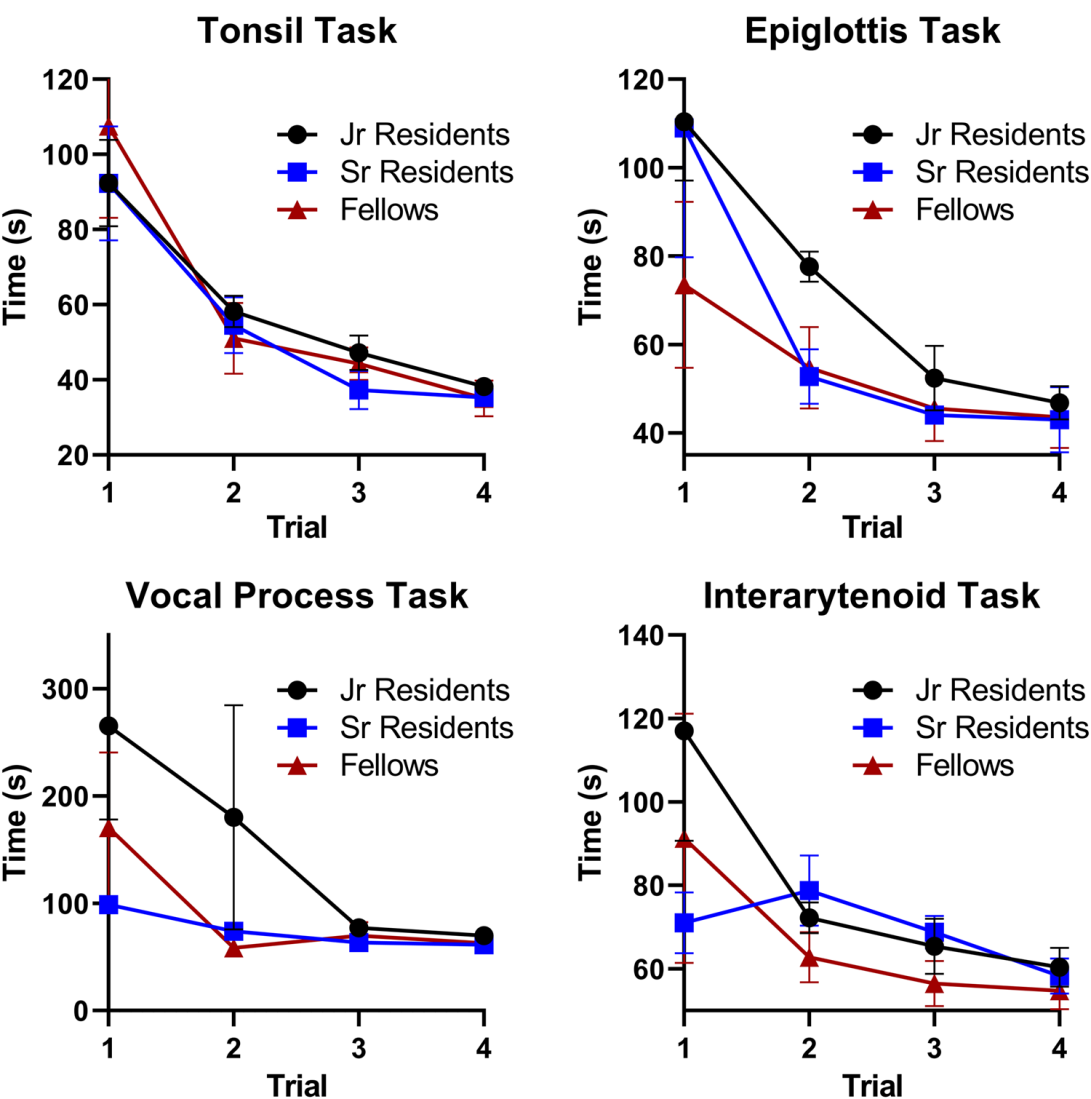
Total time for task completion by training level. For the more difficult tasks such as the epiglottis, vocal process, and interarytenoid tasks, the trainees with less experience tend to have a longer task completion time in the first trial. However, after four trials, the three groups all showed improvement and equalized

**Fig. 4:**
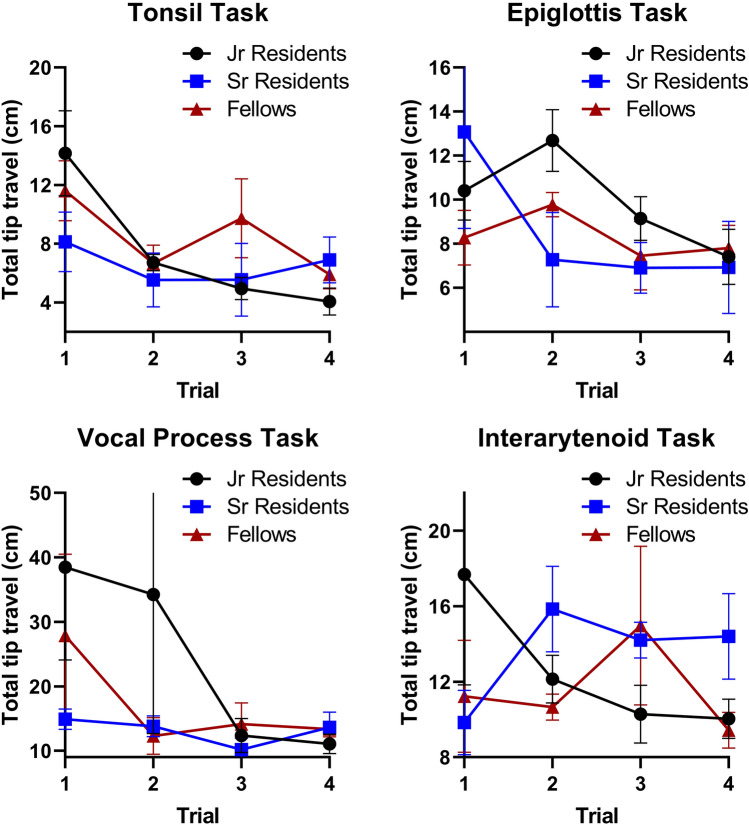
Total distance traveled for each task by training level. Similar to the previous figure, in the initial trials junior residents tend to have further distances traveled, but for most tasks all three groups improved and equalized. It is interesting to note that for the interarytenoid task, the three groups did not equalize, most likely due to the complexity of this task

5 junior residents, 4 senior residents, and 4 fellows (*n* = 13) completed the initial 3 rounds of trials. In the initial trials, there was considerable variability in task completion among the training level groups (Fig. 3). Senior residents and fellows tended to perform better initially in the tasks that required navigating to deeper aerodigestive subsites: the epiglottis, vocal process, and interarytenoid tasks. However, by the final few trials, all subjects demonstrated improvement and similar completion times regardless of training level. Similarly, the total distance traveled during task completion generally decreased over each trial with comparable distances regardless of training level (Fig. 4). In general, there were statistically significant reductions in overall time and distance traveled across the four trials for each of the four tasks (Table 1). There was no significant difference in distance traveled between the first and last trial in the epiglottis and interarytenoid tasks (using α = 0.05).

**Table 1:** Statistical analysis comparing the task completion times and total distance between the first and fourth trials

Task	1st trial time, mean (range) (sec)	4th trial time (mean, range) (sec)	*P* value	1st trial total distance (mean, range) (cm)	4th trial total distance (mean, range) (cm)	*P* value
Tonsil	97 (67–180)	36 (26–48)	< 0.001	11.5 (4.0–21.6)	5.5 (2.3–8.9)	0.002
Epiglottis	99 (36–187)	45 (27–62)	0.001	10.6 (6.2–26.1)	7.4 (5.1–11.3)	0.14
Vocal Process	185 (60–596)	65 (49–92)	0.01	29.0 (11.8–85.5)	12.6 (8.3–19.7)	0.04
Interarytenoid	95 (54–221)	58 (43–71)	0.02	13.6 (6.5–40.6)	11.2 (6.2–27.3)	0.33

In all tasks there was a statistically significant improvement in time and distance, except for the distance calculations for the epiglottis and interarytenoid tasks. Although not significant, there was still a numerical decrease in the means for these two tasks and a significantly shorter completion time

This still implies that there was an increase in velocity and efficiency for these tasks

**Fig. 5:**
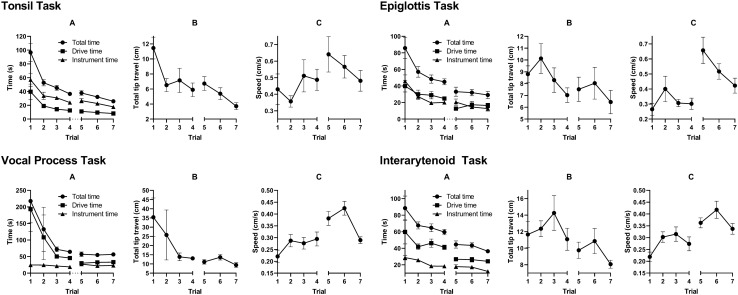
Trainee learning curve for total time (**A**), distance traveled (**B**), and speed (**C**) across four various training tasks after a 4-month gap (between trials 4 and 5). For each task, there was not a significant degradation of surgical skills after the gap, and total task completion time continued to decrease in the following trials

After a four-month interim period without usage of the device, subjects were asked to perform these same surgical tasks. 4 residents and 4 fellows (*n* = 8) were able to complete this second round of testing. Figure 5 shows the learning curves for each of the four tasks separated by time to task completion, distance traveled, and speed. These data are the compilation of the results of all residents and fellows. Between the fourth (end of first round of testing) and fifth trials (four months later), there was little to no loss of efficiency in the time to task completion, drive time, or instrument deployment time (Fig. 5A) across all four tasks. There was also a steady improvement over the next couple of trials. Similarly, the distance traveled during the drive phase (Fig. 5B) did not change substantially for each of the four tasks and continued to improve over the next two trials. Finally, endoscope speed during the drive phase did not change substantially either (Fig. 5C).

A statistical comparison was made between the fourth (end of first round of testing) and fifth (four months later) trials and is shown in Table 2. There were no statistically significant differences in the following measurements: total time, distance traveled, and speed in the tonsil task; distance traveled in the epiglottis task; total time and distance traveled in the vocal processes task; and distance traveled in the interarytenoid task. In all cases with statistical significance between the two trials, it was always an improvement, such as a reduction in total time or distance traveled or an increase in speed. It appears that the subjects continued to improve their skills with the FLEX™ Robot despite the large time gap in training.

**Table 2:** Statistical analysis comparing the task completion times and total distance between the fourth and fifth trials with a 4-month time gap between the two trials

Task	4th trial total time, mean (range) (sec)	5th trial total time, mean (range) (sec)	*P* value	4th trial total distance, mean (range) (cm)	5th trial distance time, mean (range) (cm)	*P* value	4th trial speed, mean (range) (cm/s)	5th trial speed, mean (range) (cm/s)	*P* value
Tonsil	37 (26–48)	38 (25–55)	0.73	5.9 (2.9–8.9)	6.7 (2.2–9.6)	0.39	0.49 (0.29–0.81)	0.64 (0.17–0.97)	0.19
Epiglottis	45 (27–57)	33 (20–68)	0.05	7.0 (5.1–10.7)	7.5 (4.9–13.6)	0.75	0.30 (0.18–0.49)	0.66 (0.34–1.18)	0.007
Vocal Process	65 (53–80)	57 (38–95)	0.27	13.0 (9.2–16.5)	11.1 (7.2–16.8)	0.20	0.29 (0.19–0.45)	0.38 (0.30–0.51)	0.0007
Interarytenoid	60 (43–71)	45 (28–57)	0.002	11.1 (6.8–19.8)	9.75 (5.5–13)	0.37	0.27 (0.16–0.41)	0.36 (0.30–0.46)	0.02

The majority of the tasks did not show a statistically significant difference in efficiency of operating the FLEX™ System, and all the statistically significant results showed an improvement in task completion time and distance traveled

### Clinical experience of FLEX™ robotic system

A total of 18 patients (Supplemental Table S1) were analyzed in this retrospective study with 9 males (50%), 9 females (50%), average age of 59.9 years, and an age range of 23–79 years. 6 patients (31.6%) had malignancies (T1/T2) of the head and neck. 2 patients had more than one operation within the first 20 surgical cases with the FLEX™ Robot. These operations spanned from September 2015 to October 2016. These cases were then stratified by complexity and benign versus malignant pathology.

**Fig. 6:**
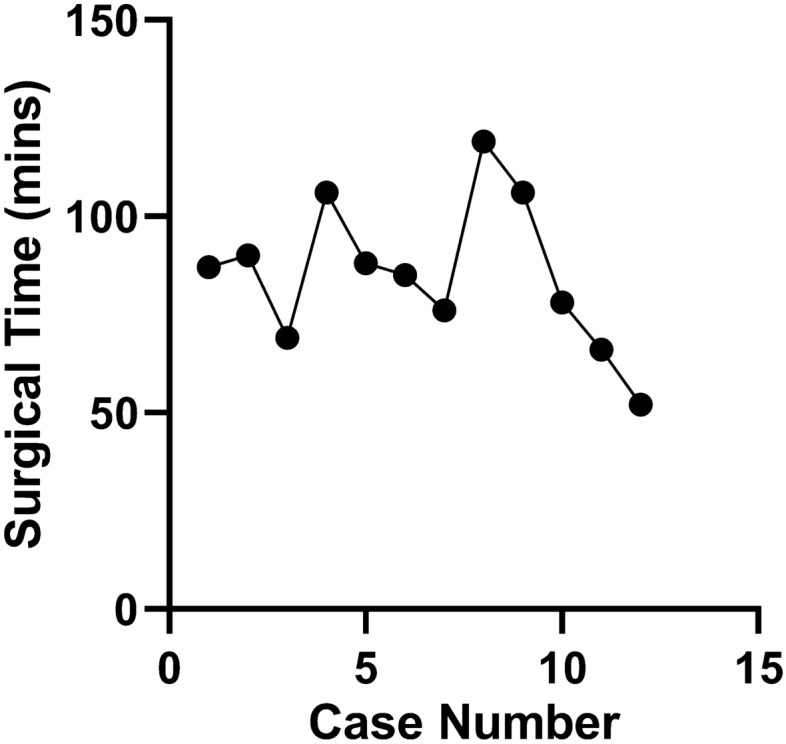
OR times for the first 12 simple benign cases using the Medrobotics FLEX™ Robotic System. These cases are a subset of the previous figure detailing the first 20 total cases. The operations spanned from September 2015 to September 2016. Although there is no clear trend for the first 7 cases due to initial limitations, a clear downward trend can be observed from cases 8 to 12 for similar consecutive operations

**Fig. 7:**
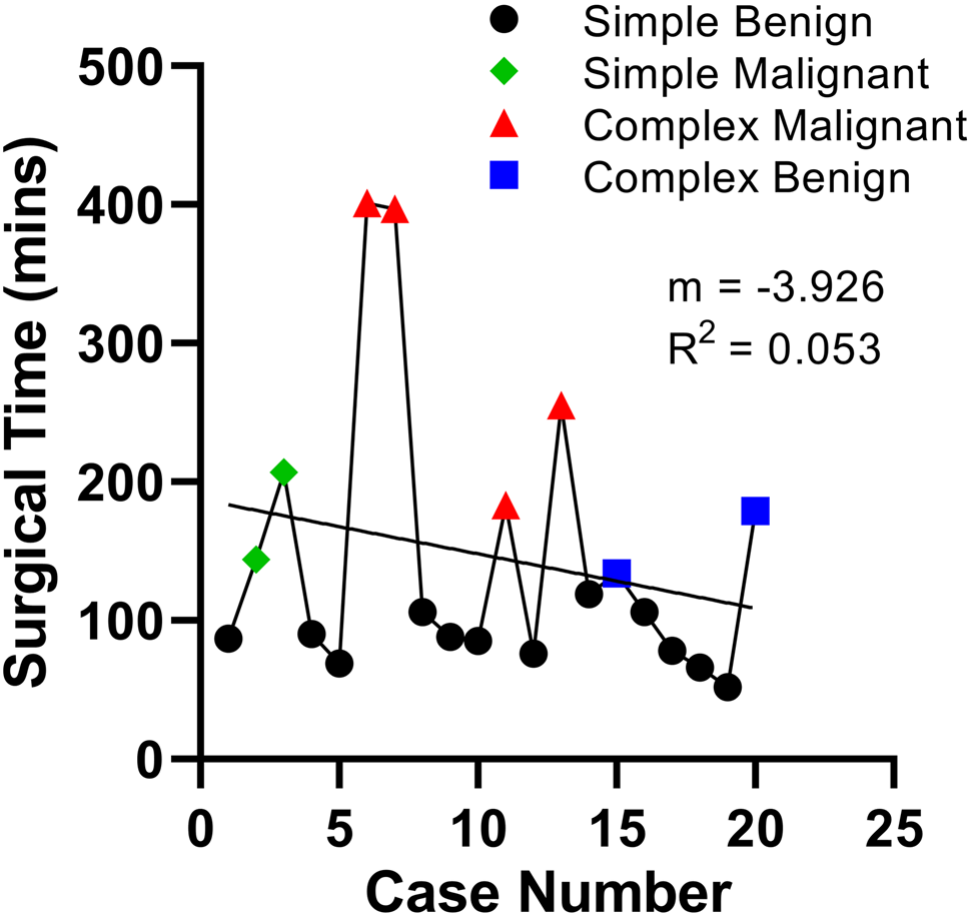
OR times for the first 20 surgical cases using the Medrobotics FLEX™ Robotic System. The operations spanned from September 2015 to October 2016. It is difficult to observe a clear trend due to a variety of factors, such as heterogeneity of case complexity, trainee turnover, and lack of availability to the true robot usage time

Among the first 12 “simple” and benign subset of cases from September 2015 to September 2016, the first 7 cases do not show any obvious changes in surgical time or downward trend (Fig. 6). However, cases 8–12 show a steady decline of time spent in the operating room from 119 to 52 min across a one-month time span (August 2016 to September 2016). However, when these data are expanded to the entire 20-case cohort, including complex operations with malignant pathology, a clear trend is difficult to observe (Fig. 7). A simple linear regression yielded a slope of − 3.926 and R square of 0.053.

## Discussion

Transoral robotic surgery is a relatively new tool in the head and neck surgery first pioneered by Weinstein and colleagues at the University of Pennsylvania [7] and gained FDA approval in 2012 [8]. Initially, the da Vinci robotic system was adopted from thoracic and abdominal surgery for TORS procedures as it has proven versatile for resection of many lesions previously accessible only via external approaches. However, this system requires placement of long, rigid devices on a rod telescope and two working arms through the patient’s mouth. In patients with small oral openings, poor neck extension, or anything more than minimal trismus, it is often impossible to safely deploy this robotic system transorally. The Medrobotics FLEX™ System was developed to address these complications of difficult anatomy and extensive oral retraction, as it contains a computer-driven, flexible endoscope that contains a camera and two working ports for instruments. These are all designed specifically for transoral usage via a single apparatus that can be deployed through the oral opening.

The adoption of any new technology has an associated learning curve, and robust analysis of the learning process of that device is crucial in its successful clinical implementation. One of the key features of the da Vinci system is its intuitive user interface, and its rapid learning curve has been previously described for a variety of surgical procedures [9–12]. In addition, the learning curve and changes in post-operative complications for transoral da Vinci use in head and neck cancers has been described [13]. Thus, in this study we investigated the ease of use and learning curve of the FLEX™ System in both training with an anatomic mannequin model and in a real-world clinical environment.

There was a wide variability among the subjects when first using the FLEX™ System in the training environment. Some subjects quickly learned to maneuver the endoscope within the upper airway and deploy surgical instruments, while others required several attempts to obtain adequate exposure, leading to long task completion times and travel distances. This prolongation was most apparent with laryngeal tasks, as they required more precise navigation of the endoscope in a long, curved path through the oral cavity and oropharynx. However, after multiple learning trials, subjects were able to rapidly gain familiarity with the system and were more efficient at deploying instruments in the upper aerodigestive tract. Although the distance traveled may not have statistically improved in the epiglottis and interarytenoid tasks, the task completion time and therefore velocity have improved for all tasks. The results from our mannequin trials suggest that the learning curve of purely operating the FLEX™ System is quite rapid and may not require a significant time investment to develop competence. It also suggests that a similar level of competence can be reached no matter the initial training level of the user.

Retention of this learning is also an important factor in clinical practice. Especially with new trainees, it is typical to have a significant time gap between the initial training on a surgical device and their first real usage of the technique in clinical practice. This potential degradation of surgical skills is important but not well understood, as there is no literature investigating this degradation in the context of TORS. There has been significant research in other traditional surgical techniques, such as laparoscopy and robotic surgery with the da Vinci system. For laparoscopy, there are mixed reports from the literature. Kahol et al. studied the degradation of surgical skills following a simulation laboratory and found that laparoscopic skills did not significantly change after 3 months but noticed that there was a steady loss of proficiency afterwards [14]. Hiemstra et al. found that following a laparoscopic training program, overall deterioration in proficiency was seen after 12 months of inactivity with some individuals not showing any degradation [15]. Alternatively, Rosenthal et al. did not find any loss of laparoscopic skills up to 12 months following a structured training program [16].

Robotic surgical skill retention with the da Vinci system following initial training similarly shows mixed results. Foell et al. found no loss of efficiency but a small increase in the number of errors in simulated robotic tasks 5 months after an initial training program [17], though only 16% of their participants completed the follow-up testing. In a larger study of 51 subjects by Jenison et al., they demonstrated a significant loss of proficiency with robotic tasks just 4 weeks after training program completion [18]. These da Vinci robotic tests included needle passing, ring transfer exercises, and other artificial tasks that, while useful and easily replicated, do not directly correlate to real-world surgical techniques, especially with a non-linear device, like the FLEX™ System used in TORS.

In this study, we assessed learning curves and retention of proficiency with the FLEX™ System 4 months after an initial training period. We found no loss of efficiency in navigation of the endoscope or instrument handling, measured by time to task completion, total distance traveled by the endoscope tip, and average speed of endoscope movement throughout the task. In fact, improvements in efficiency were noted in several measurements despite the time gap, and further improvements of the learning curves were noted after additional trials. The tasks that required the most complex navigation and setup of the FLEX™ System, such as those tasks involving larynx visualization, showed the most improvement in total time and driving speed. Simpler tasks, such as the tonsil task, that require relatively shorter and simpler navigation of the endoscope showed no significant change over the 4-month break across all measurements. This suggests that increases in performance efficiency with the FLEX™ System can be durable despite periods of non-use.

Although measuring the learning curve of using the FLEX™ System in a simulated environment is important, we also studied the clinical usage of the device. There is an expected additional learning curve here, as the operation is performed on real human anatomy with physiologic factors, such as tissue elasticity, hemodynamic considerations, and pathology. In parallel, there is a separate learning curve for the operating room staff as they become progressively more familiar with the setup and docking of the FLEX™ System.

The analysis of the first 20 clinical uses of the Flex system did not demonstrate an obvious learning curve, likely due to a variety of confounders. First, there is significant heterogeneity in the types of cases performed––initially the system was used for simple cases with benign pathology but eventually was used as a tool in complex oncologic procedures. When these factors were controlled for and sequential analogous cases were considered, a demonstrable improvement in overall OR time was encountered. Other confounders include trainee turnover and OR staff familiarity with the system. All of these factors likely impair the ability to demonstrate a smooth decrement in total OR time. More importantly, however, these factors underscore the importance of developing systematic training programs for operators and staff as any novel technology is introduced in the clinical setting.

There are important limitations of this study to be noted. The experiments using the airway mannequin were performed in a highly artificial environment that does not allow for tissue manipulation, cutting, suturing, or demonstration of more advanced surgical techniques. However, they are perhaps closer to clinical practice compared to other evaluations of robotic surgical skills on the da Vinci, such as a ring transfer exercise. The mannequin experiments primarily focused on endoscope navigation and basic use of surgical instruments, but other aspects of using the overall system, such as patient positioning, FLEX™ System setup, retractor placement, and other tasks necessary to perform TORS, were not addressed in this part of this study. In addition, the subjects for this study were composed entirely of trainees, ranging from junior residents to fellows. While this is an important group to assess acquisition and maintenance of new surgical skills, it is unclear how the learning curve results developed in this study apply to practicing attending physicians. Depending on the scope of their daily practice and familiarity with robotic and endoscopic techniques, it is possible that they could demonstrate faster or slower learning curves using the FLEX™ System compared to trainees. Thus, further assessment of this study with a broader pool of subjects that includes various attending-level physicians will be important. Incorporation of an expert surgeon would have been ideal in this study to serve as an objective, “gold standard” metric with which to compare trainee proficiency. However, we still believe that this data still demonstrates that users are able to rapidly improve performance using non-linear robotic systems.

Although we did study the usage of the FLEX™ System clinically, which addresses some of the limitations of the mannequin studies, such as tissue manipulation and robotic setup time, the overall experimental setup was not as robust in comparison. There was no magnetic tracker to precisely analyze travel distance, and due to the study’s retrospective nature, the pure robotic usage time was not captured. While total surgical time can be a potential correlate for robotic task completion time, there are numerous confounding factors present. For example, difficulty intubating, difficulty obtaining visualization, difficulty docking the robot, and submission of frozen samples for positive margins are some of the factors noted that could artificially prolong the OR time without any real effect on robotic usage. However, an argument can be made that these peri-robotic considerations are a more realistic representation of the usage of the FLEX™ System and has its separate learning curve that is expected to increase in efficiency over time. Finally, contrary to the simulated FLEX™ robot study subjects, the subjects in the clinical part of this study were specifically only one attending physician. This attending is very familiar with robotic and endoscopic surgery, so it is unclear how learning the FLEX™ System would change for attendings or trainees. There is also the consideration of different staff for each operation and swapping of trainees, which could affect overall OR time due to “refreshing” learning curves.

Despite these limitations, this study demonstrates that subjects may rapidly acquire proficiency with the Medrobotics FLEX™ System and their skills do not significantly degrade after several months of disuse. These are important considerations for future training with this system, as it suggests that a relatively short and intense training regimen may be sufficient to develop skills in trainees that remain durable for several months before their first application in the clinic. Even when used in the clinic, although there may be a separate initial learning curve due to the inherent difference between simulation and real-world application, measurable proficiency with the device can improve if and only if a proper training regimen of similar procedures is performed. Future studies will be needed to further define the optimal training regimen and safety profile to effectively bring this system into widespread clinical practice.

## Supplementary Information

Below is the link to the electronic supplementary material.

Supplemental Table 1. Patient demographics for the first 20 total cases. DL: direct laryngoscopy, BOT: base of tongue, LND: left neck dissection, RND: right neck dissection, KTP laser: potassium titanyl phosphate laser (docx 16 kb)
