# Supplementary material for: Assessing the learning curve associated with a novel flexible robot in the pre-clinical and clinical setting
Source: Surg Endosc. Author manuscript; Available in PMC 2023 Feb 1. (PMC8733873; doi:10.1007/s00464-021-08445-7)
Supplement: Supplementary file 1 [file NIHMS1764484-supplement-Supplementary__Materials.docx]

| Case Number | Patient Number | Gender | Age | Procedure Performed | Classification |
| --- | --- | --- | --- | --- | --- |
| 1 | 1 | F | 57 | Partial glossectomy with resection of BOT tumor | Simple benign |
| 2 | 2 | M | 57 | DL and biopsy of R BOT tumor | Simple malignant |
| 3 | 3 | M | 70 | DL, robotic resection of left hypopharyngeal lesion | Simple malignant |
| 4 | 4 | F | 23 | Resection of lingual thyroid and BOT | Simple benign |
| 5 | 5 | M | 64 | Resection of supraglottic lipoma | Simple benign |
| 6 | 6 | M | 79 | DL, supraglottic laryngectomy, LND (IIa-IV), RND (IIa-IV), feeding tube placement | Complex malignant |
| 7 | 7 | M | 77 | DL, L hemiglossectomy, left BOT resection, LND (I-IV), left parotidectomy | Complex malignant |
| 8 | 8 | F | 63 | DL and left false cord resection | Simple benign |
| 9 | 9 | F | 56 | DL and debulking of epiglottic lesion (laryngeal surface) | Simple benign |
| 10 | 10 | M | 56 | DL and bilateral laryngocele resection | Simple benign |
| 11 | 11 | F | 58 | Supraglottic laryngectomy, DL, and esophagoscopy | Complex malignant |
| 12 | 12 | F | 68 | Transoral resection/reconstruction, incisional biopsy of vellecular lesion | Simple benign |
| 13 | 13 | F | 78 | DL with microsuspension and supraglottic laryngectomy | Complex malignant |
| 14 | 6 | M | 79 | Resection of left arytenoid and left epiglottectomy with airway dilation | Simple benign |
| 15 | 14 | M | 42 | DL, microlaryngoscopy, removal of right false vocal fold lesion | Complex benign |
| 16 | 5 | M | 64 | Micro DL and resection of left supraglottic lipoma | Simple benign |
| 17 | 15 | M | 47 | DL, microsuspension laryngoscopy, robotic assisted KTP laser ablation of hemangioma of tongue base | Simple benign |
| 18 | 16 | F | 62 | Supraglottic laryngectomy | Simple benign |
| 19 | 17 | M | 59 | Transoral resection of laryngeal lesion | Simple benign |
| 20 | 18 | F | 61 | Bilateral lingual tonsillectomy, DL and adenoidectomy, removal of right posterior mandibular molar | Complex benign |
|  |  | M – 9 (50%) F –  9  (50%) | Mean: 59.9 ± 14.3 |  |  |

**Supplemental Table 1**
